# MAPfastR: Quantitative Trait Loci Mapping in Outbred Line Crosses

**DOI:** 10.1534/g3.113.008623

**Published:** 2013-10-11

**Authors:** Ronald M. Nelson, Carl Nettelblad, Mats E. Pettersson, Xia Shen, Lucy Crooks, Francois Besnier, José M. Álvarez-Castro, Lars Rönnegård, Weronica Ek, Zheya Sheng, Marcin Kierczak, Sverker Holmgren, Örjan Carlborg

**Affiliations:** * Department of Clinical Sciences, Swedish University of Agricultural Sciences, SE-75007 Uppsala, Sweden; † Department of Animal Breeding and Genetics, Swedish University of Agricultural Sciences, SE-75651 Uppsala, Sweden; ‡ Department of Information Technology, Uppsala University, SE-75105 Uppsala, Sweden; § Department of Cell and Molecular Biology, Uppsala University, BMC SE-75124 Uppsala, Sweden; ** Welcome Trust Sanger Institute, Hinxton, Cambridge, CB10 1HH, United Kingdom; †† Department of Genetics, University of Santiago de Compostela, ES-27002 Lugo, Galiza, Spain; ‡‡ Instituto Gulbenkian de Ciência, Evolutionary Genetics Group, Oeiras, Portugal; §§ Statistics Unit, Dalarna University, SE-791 88 Falun, Sweden; *** State Key Laboratory for Agro-Biotechnology, China Agricultural University,100193, Beijing, People’s Republic of China

**Keywords:** QTL mapping, inbred line cross, outbred line cross, quantitative genetics

## Abstract

MAPfastR is a software package developed to analyze quantitative trait loci data from inbred and outbred line-crosses. The package includes a number of modules for fast and accurate quantitative trait loci analyses. It has been developed in the R language for fast and comprehensive analyses of large datasets. MAPfastR is freely available at: http://www.computationalgenetics.se/?page_id=7

Quantitative trait loci (QTL) mapping is a valuable tool for unraveling the complex genetic architecture of phenotypic traits. A number of software packages currently are available for detecting QTL from marker data (for reviews, see Manly and Olson 1999; Durrant *et al.* 2011; Zhou *et al.* 2012). Most of the software were developed for analyses of various types of crosses between inbred lines, including backcrosses and F2 crosses (R/QTL, Broman 2003; Joehanes and Nelson 2008), multicross designs (heterogeneous stocks and collaborative crosses; Mott *et al.* 2000; Jourjon *et al.* 2005; Huang and George 2011), and advanced intercross lines (Peirce *et al.* 2008). Although we have written an extension to R/QTL that enables data from outbred lines to be analyzed (Nelson *et al.* 2011), the functionality is limited. There is some software designed for outbred populations and line crosses (*e.g.*, QxPak, Pérez-Enciso and Misztal 2004 and GridQTL, Seaton *et al.* 2006), but as they are several years old, and the algorithms they use are not able to handle the large amount of data produced by current SNP chip technology (Crooks *et al.* 2011).

MAPfastR is a fast and comprehensive software package for analyzing QTL data from outbred line-crosses that has been developed for flexible analyses of large datasets. MAPfastR is distinct from other packages in several ways. Notably, MAPfastR is based on a computationally efficient algorithm that uses all available data from dense SNP-chips (*i.e.*, tens to hundreds of thousands of markers, similar to association studies) and pedigree information (Crooks *et al.* 2011). MAPfastR provides functionality for F_2_ crosses and backcrosses under the assumption that different QTL alleles are fixed in the founder lines (Crooks *et al.* 2011), line-cross analyses allowing for within-line segregation (flexible interclass analysis [FIA]; Rönnegård *et al.* 2008), and tests for epistatic interactions (Carlborg and Andersson 2002). In addition to the standard functionality, the software comes with add-on packages that allow more experienced users to take advantage of modules for analyses of deep (Advanced Intercross Line) pedigrees. MAPfastR includes an online developer and community-based support system. MAPfastR is implemented in the R language (with optimization of the more computationally intensive algorithms in C++), accepts several standard input formats and is available for Windows, Unix, and Mac OS.

## Implementation

**Figure 1:**
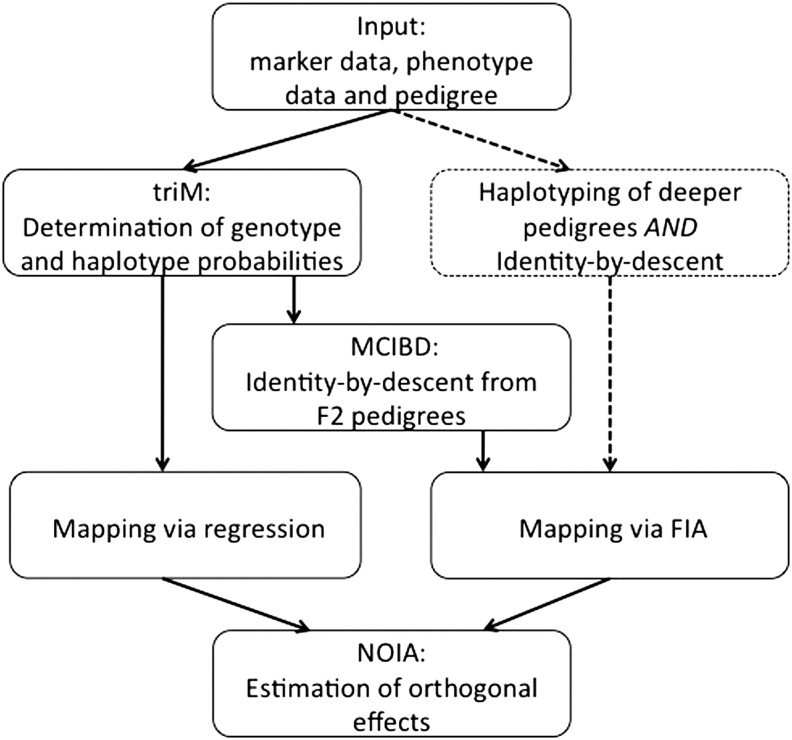
Outline of MAPfastR.

MAPfastR integrates a number of published analytical tools by providing them within a comprehensive R package with a user-friendly interface and accompanying documentation. An outline of the analysis pipeline is shown in Figure 1. The main functions in the package are briefly described herein.

### Data import

The first release (v 1.0) of MAPfastR supports two major input formats (CRI-MAP, Green *et al.* 1990 and triM, Crooks *et al.* 2011). All imported data are stored in a standardized R object, which consists of a list with two main components (phenotypic and pedigree data, and genotypic data) and several optional attributes (storing, for example, information on which is the heterogametic sex and which is the sex chromosome). A full description of the data object is provided in Supporting Information, File S1. Once the data import is completed, the internal data format can be used for outbred pedigree analyses by use of the main and supplementary analysis modules as well as additional custom analyses coded by the user. As output from the available functions is produced, it is appended to the data object, facilitating further analyses using the results. Support for more formats is in progress and will be provided in coming releases.

### Least-squares QTL mapping

A module is provided to perform QTL mapping by least-squares regression (Haley and Knott 1992), where a user-selected phenotype is regressed onto genetic effect variables derived from genotype probabilities.

#### Calculation of QTL genotype probabilities using trim:

The probabilities of alleles in the mapping population originating from each founder line are calculated using the triM algorithm (Nettelblad *et al.* 2009; Crooks *et al.* 2011). The algorithm uses a hidden Markov model to trace allele transmission in the pedigree. The line origins are calculated at user-defined, regular intervals along the chromosome and returned to the data object for further analysis.

#### Regression analysis:

Estimates of QTL effects are provided together with a plot of the test statistic from the fitted model across the genome, which illustrates the QTL locations. Analyses can be done on both autosomes and the homogametic sex chromosome, and for backcrosses and F2 crosses. Permutation testing can be performed by creating appropriately permuted datasets to derive an empirical significance threshold (Churchill and Doerge 1994).

### FIA

FIA (Rönnegård *et al.* 2008) is an algorithm developed for analyses of outbred line-cross data where it is not reasonable to assume fixation of different QTL alleles in the founder lines. The analysis is performed by the following two steps.

#### IBD estimation using MCIBD:

Identity by descent (IBD) matrices are estimated from the QTL genotype probabilities calculated from triM using the Monte Carlo Identity-By-Descent Matrix Estimation (MCIBD) algorithm (Shen *et al.* 2011). These matrices are used in the second step of the FIA analysis.

#### FIA:

The variance-component-based FIA analysis scans the genome and provides estimates of genetic effects at regular spaced, user-defined locations in the genome as well as estimates of the likelihood that the QTL is fixed or segregating within the founder lines. The significance testing is based on a score-statistic and empirical significance thresholds are derived by permutation (Rönnegård *et al.* 2008).

### Estimation of genetic effects using the Natural and Orthogonal InterAction model

The Natural and Orthogonal InterAction (NOIA) model is a unified model that ensures genetic effect estimates are orthogonal and enables effects to be translated from one population to another, aiding biological interpretation (Álvarez-Castro and Carlborg 2001). This allows users to estimate, for example, interaction effects that are comparable between populations and construct high-order genotype-phenotype maps for further analyses of interactions (*e.g.*, Álvarez-Castro and Rouzic 2008; Le Rouzic *et al.* 2008; Le Rouzic and Álvarez-Castro 2008).

### Variance-component-based analysis of deep intercross pedigrees

An external module for performing analyses of deep pedigrees is provided as an unsupported add-on function for advanced users (Besnier *et al.* 2011). When this module is used, individuals from Advanced Intercross Lines generated from outbred founders can be haplotyped and an IBD matrix created that can be used to screen the genome for QTL using the FIA module for variance-component-based analysis.

## Results

Each of the functions has been extensively tested during development (Crooks *et al.* 2011; Ek *et al.* 2012; Shen *et al.* 2012). The complete pipeline has also been thoroughly tested to ensure that the package performs as a whole. Sample code for a complete analysis of an outbred line-cross and the resulting output is available in the supplementary documentation and example files.

In conclusion, MAPfastR is a comprehensive, fast, and accurate software that is able to perform various methods for QTL mapping in outbred line-cross data. It can also be used for analyzing data from inbred line crosses, where the computational efficiency may be a benefit. Add-on functions for the analysis of deeper pedigrees are also provided for advanced users. MAPfastR is under ongoing development to extend and improve its functionality and is extensively documented, with support available through an online forum for community and developers alike (https://groups.google.com/d/forum/mapfastr).

## Supplementary Material

Supporting Information
