# Supplementary material for: MAPfastR: Quantitative Trait Loci Mapping in Outbred Line Crosses
Source: G3 (Bethesda). 2013 Oct 11;3(12):2147–9. doi: 10.1534/g3.113.008623 (PMC3852377; doi:10.1534/g3.113.008623)
Supplement: Supporting Information [file supp_3_12_2147__index.html]

MAPfastR: Quantitative Trait Loci Mapping in Outbred Line Crosses — Supporting Information 

# MAPfastR: Quantitative Trait Loci Mapping in Outbred Line Crosses

## Supporting Information for Nelson *et al.*, 2013

**Files in this Data Supplement:**

- File S1 - MAPfastR Package Tutorial (PDF, 1 MB)
