# Supplementary material for: MAPfastR: Quantitative Trait Loci Mapping in Outbred Line Crosses
Source: G3 (Bethesda). 2013 Oct 11;3(12):2147–9. doi: 10.1534/g3.113.008623 (PMC3852377; doi:10.1534/g3.113.008623)
Supplement: Supporting Information [file supp_g3.113.008623_008623SI.pdf]

G3 Genes | Genomes | Genetics

MAPfastR: QTL mapping in outbred line crosses

Ronald M. Nelson[*,§]
Carl Nettelblad[†]
Mats E. Pettersson[*,§]
Xia Shen[*,‡]
Lucy Crooks[§]
Francois Besnier[§]
José Álvarez-Castro[§]
Lars Rönnegård[**]
Weronica Ek[§]
Zheya Sheng[*,§,§§]
Marcin Kierczak[*,§]
Sverker Holmgren[†]
Örjan Carlborg[*,§]

[*]Department of Clinical Sciences, Swedish University of Agricultural Sciences, SE-75007 Uppsala, Sweden.
[§]Department of Animal Breeding and Genetics, Swedish University of Agricultural Sciences, SE-75651 Uppsala, Sweden.
[†]Department of Information Technology, Uppsala University, SE-75105 Uppsala, Sweden.
[‡]Department of Cell and Molecular Biology, Uppsala University, BMC SE-75124 Uppsala, Sweden
[**]Statistics Unit, Dalarna University, SE-78170 Borlänge, Sweden
[§§]State Key Laboratory for Agro-Biotechnology, China Agricultural University,100193, Beijing, People's Republic of China

Software available at: http://www.computationalgenetics.se/?page_id=7

Corresponding author:
Ronald M Nelson
Swedish University of Agricultural Sciences
Department of Clinical Sciences
Box 7078
Ulls väg 24E
SE-75651 Uppsala
Sweden
+46700719244
ronnnie.nelson@slu.se

DOI: 10.1534/g3.113.008623

File S1

MAPfastR Package Tutorial

Ronald M. Nelson, Xia Shen, Marcin Kierczak,
Mats Pettersson, Zheya Sheng, Örjan Carlborg

August 5, 2013

Abstract

MAPfastR is an R package for mapping quantitative trait loci (QTL) in outbred line crosses. It contains all the functions required to perform a QTL analysis in outbred lines. Some of the functions implement different algorithms that can be used interchangeably and it is up to the user to decide which will be applied. This gives the user a certain degree of flexibility when constructing the analyzes pipeline and helps creating custom-tailored solutions depending on the type of analyzed data and the aims of the research.

This document is a tutorial that aims at presenting all the basic aspects of working with the package by presenting a complete analysis workflow.

Introduction

MAPfastR is a software package developed to analyze QTL data from outbred line-crosses. It has been developed for fast and comprehensive analyses of large datasets implemented in the R language. The package has been developed for flexible analyses of large datasets. It includes an online developer and community-based support system. MAPfastR is based on a computationally efficient algorithm that uses all available data from dense SNP chips [1]. MAPfastR provides functionality for F2 crosses and backcrosses under the assumption that different QTL alleles are fixed in the founder lines [1], line-cross analyses allowing for within line segregation [2] and tests for epistatic interactions [3]. In addition to having standard functionality, the software comes with add-on packages that allow more experienced users to take advantage of modules for analyses of deep (Advanced Intercross Line) pedigrees. MAPfastR is implemented in the R language (with optimization of the more computationally intensive algorithms in C++); it accepts several standard input formats and is available for Windows, UNIX, and Mac OS.

Support

Users' forum is available at https://groups.google.com/d/forum/mapfastr. There you can post your questions and opinions and get the newest information directly from package developers.

Some useful information you can access via Computational Genetics Group web site at http://www.computationalgenetics.se.

Aim of this tutorial

This tutorial brings provides a complete example workflow. Not all the possible analyses are documented here because numerous variations in analyses can be performed. The different parameters and other modules are available in the Help documentation in R. Additionally, a number of modules for more specialized analyses are still under development and not included in this tutorial. However, the tutorial provides a complete workflow for all the main analysis components for a QTL mapping study in outbred line cross data.

This tutorial will be updated in response to the feedback from the users and as the new functionalities are added. Please keep track of new releases to make sure you have the latest version. An overview of the package structure is presented in Figure 1.

Installation

Before using MAPfastR, the package has to be installed in the R environment. This can be accomplished entering the following single line*:

```
install.packages('MAPfastR',
repos='http://www.computationalgenetics.se/MAPfastR')
```

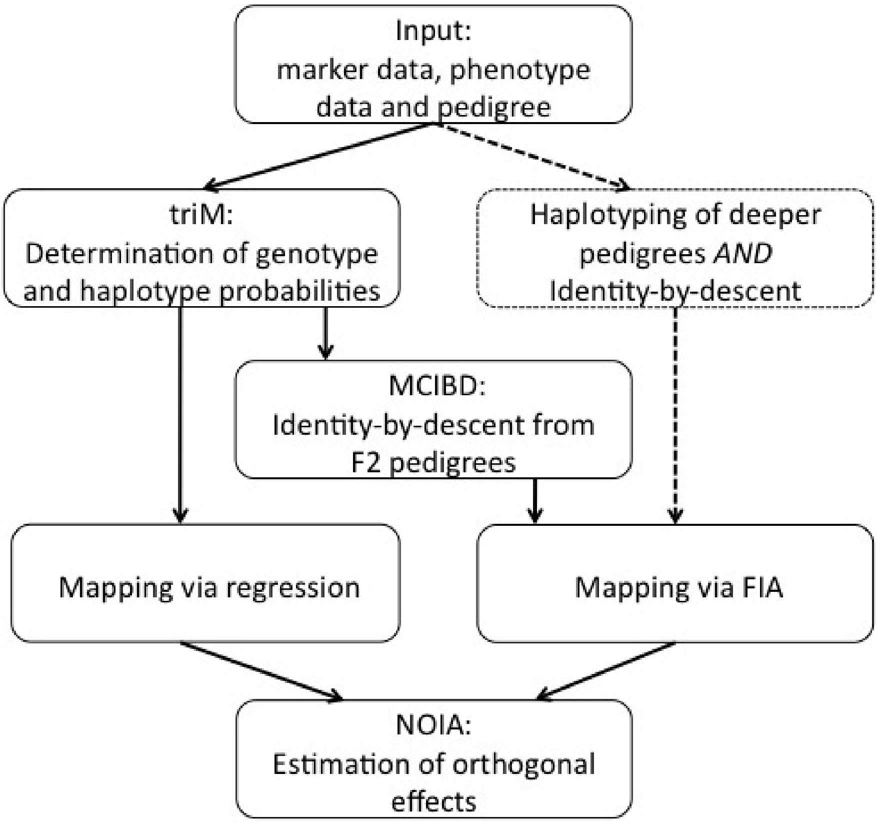

Figure 1: A diagrammatic representation of the package. Dashed arrows represent modules under development that are not covered in this tutorial.

Note: This installation is for the Windows and OS X binaries. R version 3.0.0 is required (with limited support on R version 2.15.3). It is also possible to install the package and all the dependencies from source (see the information at the end of the document).

To run MAPfastR a number of other dependencies need to be installed. These should install automatically and includes hglm, Matrix, lattice, MASS, dglm, stat mod, Rcpp, noia, cnf2freq, cnf2freqibdtracer, sfsmisc, snow and snowfall. Once the package is installed, it has to be loaded into the R environment:

```
library("MAPfastR")
```

One can list the vignettes in the package and open this document by

```
vignette(package = "MAPfastR")
vignette("MAPfastR")
```

The package needs to be loaded every time, a new R session is started!

At any time, general help in R can be obtained by typing

```
help.start()
```

Importing Data

Once the package is loaded and ready to use, you have to load the data. We have prepared an example dataset that will be used throughout this tutorial. The following section describes data formatting in a more detailed way. Please note that MAPfastR package accepts data in two existing and popular formats: cnf2freq/triM format and CriMap format. Below is a description of the triM format and we encourage you to consult the documentation CriMap for more details on its file format. The data in either of the supported formats can be directly imported to MAPfastR package using the `import_data` function as described below.

Example data

This tutorial uses a simple example dataset that is a subsection of an F2 cross between two chicken breeds. It consists of two chromosomes, neither of which is a sex chromosome. The phenotype "SC" in the example is the shank circumference for each individual. The data is stored in four files in triM format; the data can be found in the MAPfastR folder in the package installation directory.

- `marker_test.txt` – file containing genotyping data
- `mrkinfo_test.txt` – file containing information about markers
- `ped_test.txt` – file containing pedigree information
- `pheno_test.txt` – file containing phenotypes and co-variates, e.g. sex

Data format

The triM format is used in the example files. Note that all the files are tab delimited.

The marker information files:

The first line in the marker information files provide information on the number of chromosomes and the total number of markers. The following lines, in the marker information file, contain the total number of markers per chromosome (first column) followed by a numeric ID that indicates which column pair in the genotype file contains the genotypes for that marker. This information is given on one line for each chromosome. After this block of lines, the space between each marker is provided (i.e., the map data), also one line for each chromosome. The last block of lines contains the name of each marker, one line for each

chromosome. Note that the first column of each row containing the positions and names of the markers should be filled with a '1'.

The example marker information file: "mrkinfo_test.txt"

The genotype file:

The genotype file provides the genotypic information for each individual. The individual IDs are indicated in the first column of each row. The integer values in the following columns indicate the genotype at each locus in sequence. Every 2 columns correspond to one marker (i.e., one allele in each column) and the columns are arranged sequentially in the order described in the marker file.

The example marker information files: "marker_test.txt"

The pedigree file:

The pedigree file is arranged in full-sib families. It provides the number of F2 individuals within each family. The first column has the individual IDs (starting with the F0 generation, then the F1s and then the F2s). The next two columns contain the ID of each individual's parents IDs followed by the individual's sex. Note: for the F0 generation the parents are indicated with a '0' and an additional column with line origins is provided.

The example pedigree file: "ped_test.txt"

The phenotype file:

The phenotype files provide the information on the phenotypes of all the individuals. In the first line, the first column contains the heading "ID", followed by the phenotype names. The columns are filled with first the individual IDs and then the phenotypic values, as indicated by the headings.

The example pedigree file: "pheno_test.txt"

Importing the example data

Prior to importing the data, the user has to change the working directory to the folder where the data is found. This can be done with the function `setwd(dir)` where "dir" indicates the directory with the data files. For example, on a standard Windows installation the following command will change the working directory the correct folder for the following input command to be used directly:
`setwd("C:/Users/user_name/Documents/R/win-library/3.0/")`

Note: The directory may be slightly different for each user.

Once the working directory points to where the files are located the data can be imported with the following lines:

```
mydata <- import_data(pedigree_file = "ped_test.txt",
                      phenotype_file = "pheno_test.txt",
                      genotype_file = "marker_test.txt",
                      marker_file = "mrkinfo_test.txt",
                      n_gen_map = 1,
                      sex.chrom=NA)
```

The `mydata` variable contains a data object in an internal MAPfastR format. The data object is a list with two main data frames. The first contains all the phenotypic as well as the pedigree information. The second contains the genotypic and the genetic map information. After the two data frames, auxiliary variables are added and include information on the type of cross and the sex chromosome system of the study organism. Find the variable names of the data object by entering the following lines:

```
# Show variable names inside my data object
names(mydata)
[1] "pheno"              "geno"              "backcross"
[4] "backcross.line"     "backcross.parent"  "sex.chrom"
[7] "heterogam"          "sex.restrict"
# geno itself is a data structure
# here we show only 5 last names
tail(names(mydata$geno), 5)
[1] "92140"    "chr"      "sex_1_cM" "sex_2_cM" "ref_cM"
# if we want to display unique chromosomes
unique(mydata$geno$chr)
[1] 1 2
```

Calculating line origin probabilities

Calculations of genotype and phenotype probabilities are done using the triM algorithm implemented in the cnf2freq package. For details see[4] and [5]. To calculate line origin probabilities, we use the `calc.probs` function; it will add the calculated probabilities as extra columns to the existing data structure.

```
mydata <- calc.probs(data = mydata, interval = 1.0)
```

The package asks which type of analysis you will perform using the calculated probabilities. You start with regression models, so enter "1".

```
Are you calculating probabilities for:
(1) Regression models or (2) Variance component models?
1:1
```

Once the genotype probabilities are calculated, you can perform a QTL scan using the Haley Knott regression. In the following line “SC” is specified as the phenotype and with “sex” as covariate.

```
testscan <- autosome.genome.scan("SC", data = mydata, covariates = "sex")
The results for the starting model fitting sex to SC in mydata dataset are:

          Estimate Std. Error t value  Pr(|t|)
Intercept   4.6640     0.1878  24.840 8.426e-89
sex        -0.4319     0.1855  -2.328 2.033e-02

F value 5.419 on 1 and 491 degrees of freedom
```

Once the scan is completed, the results are stored in the “testscan” object:

```
# See what is the structure of the
# object returned by scan
names(testscan)
[1] "max.F.value"
[2] "position.max.F"
[3] "estimated.effects.max.F"
[4] "variables.in.starting.model"
[5] "all.F.values"
[6] "chrom.boundaries"
[7] "results.by.chromosome"
```

The scan generates F values, the test statistic from the Haley Knott regression, for each interval. It automatically produces a plot of the F values across the whole genome, (see Figure 2). To see all the information enter “testscan”.

NOIA

Finally, the Normal and Orthogonal Interactions model [6] implemented in the “NOIA” package [7] can be used to check for interactions between loci. First, choose some candidate loci by evaluating the QLT scan results. You will see two peaks on chromosome 1: one around 10-11cM, another around 19-20cM. You can retrieve their positions from the vector of markers.

```
# Select chromosome 1
ref_cM.chr1 <- mydata$geno$ref_cM[which(mydata$geno$chr == 1)]
#
# Which F-values were above 6?
myFvals <- testscan$results.by.chromosome$chrom.1$F.values
names(myFvals[myFvals > 6])
[1] "10 cM" "11 cM" "19 cM" "20 cM"
```

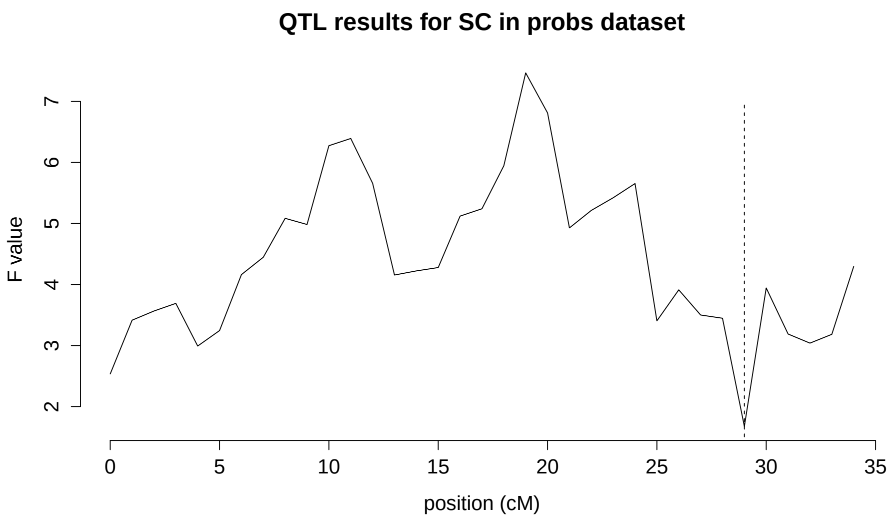

Figure 2: An example scan result. Dashed line denotes chromosome border.

```
#
# Define 2 peaks
peak1 <- which(ref_cM.chr1 > 10 & ref_cM.chr1 <= 11)
peak2 <- which(ref_cM.chr1 > 19 & ref_cM.chr1 <= 20)
```

Now, select only 2 loci (for demonstration purposes), one from each peak and try to fit linear NOIA.

```
 # Select one (the first) locus from each peak
 mymarkers <- c(peak1[1], peak2[1])
 #
 # Run NOIA
 noia_result <- run_NOIA(data = mydata, phenotype = "SC",
                markers = mymarkers, noia_func="linear")
noia_result

Phenotype:
        n= 493  min:  3.2  max:  5.1  mean:  4.2284
Genotype:
        n= 493 , 2 loci
                Locus 1              1: 0.819        2: 0.168        3: 0.0122
                Locus 2              1: 0.415        2: 0.441        3: 0.144

       Effects   Variances Std.err Pr(|t|)
..  4.2318e+00  0.0000e+00  0.0176  < 2e-16 ***
a. -3.0998e-03  1.7287e-06  0.0555  0.95551
d.  3.6912e-01  5.0866e-03  0.1609  0.02217 *
.a -2.4486e-02  2.9131e-04  0.0267  0.35970
aa -4.1243e-02  2.0884e-04  0.0778  0.59623
```

```
da -2.7147e-01  2.2152e-03  0.1800  0.13211
.d -1.9931e-02  8.6310e-05  0.0379  0.59915
ad -4.0508e-02  9.2991e-05  0.0934  0.66483
dd          NA          NA      NA       NA
---
Signif. codes:  0 '***' 0.001 '**' 0.01 '*' 0.05 '.' 0.1 ' ' 1

Variances
        Total (phen)        0.13781
        Residual        0.13471
        Explained        0.0030975        (2.25%)
        Genetic         0.007983
```

Variance component QTL analysis & FIA

The MAPfastR package provides functions for the identity-by-descent (IBD) matrix estimation and variance component QTL analysis, where the latter includes the flexible intercross analysis (FIA) [2]. As an extension of an ordinary variance component QTL analysis, FIA allows estimation of within-line segregation.

Another way to import the data into an object in this example is via (this is the same data used thus far in the tutorial):

```
data(mydata)
```

Now rerun the `calc.prob` function to get probabilities for IBD estimation. You do not need to assign the function call to a data object. An object named `output` will be created automatically.

```
calc.probs(data = mydata, interval = 100)
```

Because it takes longer to calculate probabilities for variance component analysis than for regression models, in this example the interval is set to every 100cM rather than every 1cM.

Note: Using a 2.66 GHz, Intel Core i7 Macbook Pro with 8GB RAM, this calculation can take up to 20 minutes.

When the package asks which type of analysis you will perform using the calculated probabilities, enter "2".

```
Are you calculating probabilities for:
(1) Regression models or (2) Variance component models?
1:2
```

An object `output` is created in the working memory space. Now use the MCIBD routine (see package vignette "MCIBD" for details) to calculate IBD matrices across the genome.

```
MCIBD.genome(data = mydata, trim.output = output, mc.size = 9)
```

The default setting is 99 Monte Carlo imputes for IBD estimation. You can increase this number to gain precision, and here, as an example, it is decreased to gain efficiency.

Note: Using a 2.66 GHz, Intel Core i7 Macbook Pro with 8GB RAM, this calculation can take up to 5 minutes.

After the function call of MCIBD, two folders "chr1" and "chr2" will be created in the working directory because this example dataset has only two chromosomes. In each folder, the IBD matrix at position index i is stored as "i.ibd.RData". Now you can run an ordinary variance component QTL scan for trait "SC" by entering:

```
VC.result <- FIA.scan(mydata, "SC", fixed.effects = "sex",
chr = 1:2, figure.file = "VC_scan.pdf")
```

The function call returns the FIA scores of each tested locus into `VC.result` and produces a figure for each chromosome in `VC_scan.pdf` (Figure 3; the pdf-file is created in the working directory). Furthermore, you can do a FIA scan by considering within-line correlation instead of assuming no correlation within each parental line. To do this, calculate another set of IBD matrices assuming within-line fixation. The `MCIBD` documentation contains a detailed description of how this should be defined. Briefly, for this step of FIA you only need to know how many males and females are in the founders, since within-line fixation is assumed. Enter:

```
 sum(mydata$pheno$generation == 1 & mydata$pheno$sex == 1)
[1] 14
 sum(mydata$pheno$generation == 1 & mydata$pheno$sex == 2)
[1] 19
```

The new `MCIBD.genome` function call is written as:

```
MCIBD.genome(data = mydata, trim.output = output, mc.size = 9,
segregation = rep(1:2, c(14*2, 19*2)))
```

After this function call of `MCIBD`, a new set of IBD matrices are stored in the two folders "chr1" and "chr2" as "i.ibd1.RData" for each position with index i. Now you can perform the FIA scan by setting the argument `estimate.ro = TRUE`.

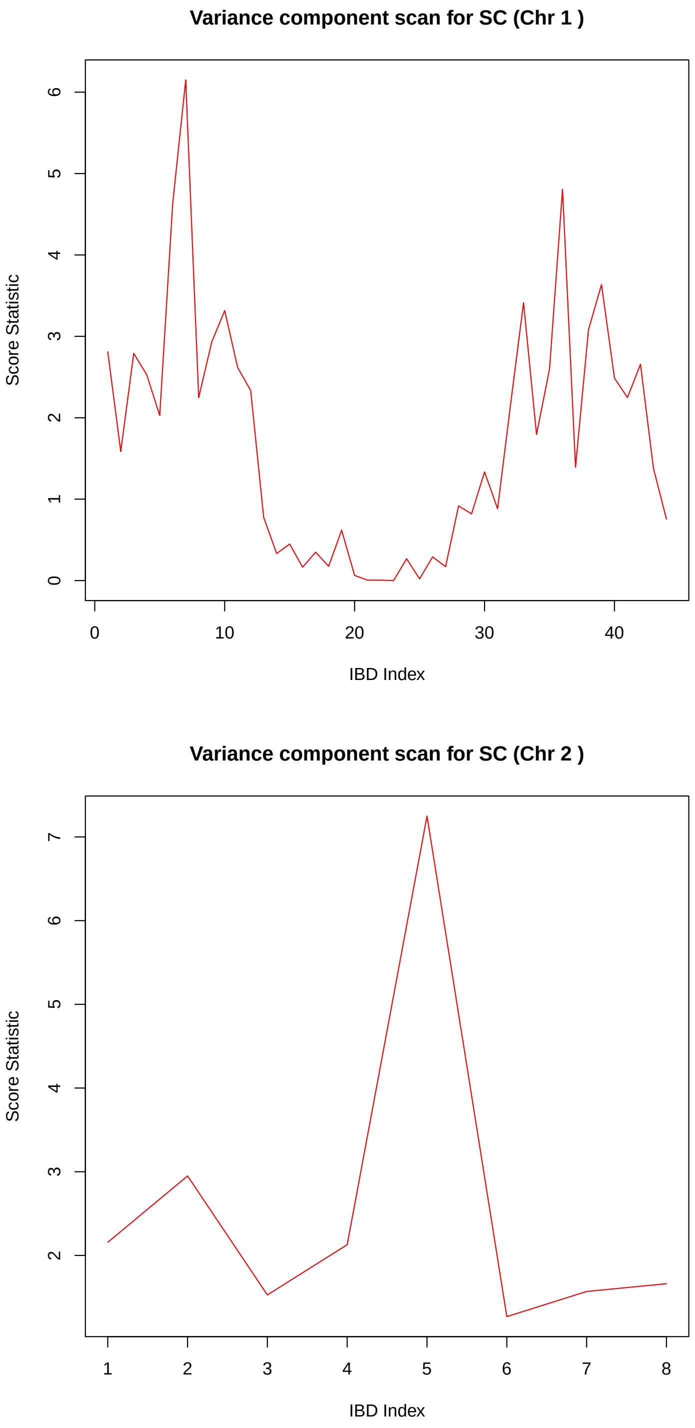

Figure 3: An example variance component QTL scan result.

```
 FIA.result <- FIA.scan(mydata, "SC", fixed.effects = "sex",
 chr = 1:2, estimate.ro = TRUE, figure.file = "FIA_scan.pdf")
```

The function call returns the FIA scores of each tested locus into `FIA.result` and produces a figure for each chromosome in `FIA_scan.pdf` (Figure 4). You will see that position 38 has a high score in the scan, therefore, you can try to fit an FIA model for this locus to estimate the within-line correlation.

```
 model <- FIA.model(mydata, "SC", fixed.effects = "sex",
 chr = 1, IBD.index = 38)
Estimated within-line correlation: rho = 0.8554861
```

You get a within-line correlation estimate of 0.91, which is close to fixation at this particular example locus. The fitted `hglm` object and the within-line correlation estimate ρ (`rho`) are returned to model, so that the generic functions such as `summary` can be applied to check the fitted random effects model. This summary directly applies to the fitted object from the `hglm` package, so for detailed explanation of each part of the summary, refer to, *e.g.*, `help('hglm')`.

```
 summary(model$model)
Call:
hglm.default(X = X, y = y, Z = cbind(L1, L2),
 conv = 1e-06, RandC = rep(length(y), 2))

----------
MEAN MODEL
----------

Summary of the fixed effects estimates:

                                                     Estimate Std. Error
(Intercept)                                            4.7055     0.2087
data$pheno[data$pheno$generation == 3, fixed.effects]  -0.4569     0.1952
                                                     t-value Pr(>|t|)
(Intercept)                                          22.548   <2e-16 ***
data$pheno[data$pheno$generation == 3, fixed.effects] -2.341   0.0197 *
---
Signif. codes:  0 *** 0.001 ** 0.01 * 0.05 . 0.1  1
Note: P-values are based on 470 degrees of freedom

Summary of the random effects estimates:

    Estimate Std. Error
Z.1   0.0053     0.0621
Z.2  -0.0405     0.0541
Z.3  -0.0403     0.0543
...
```

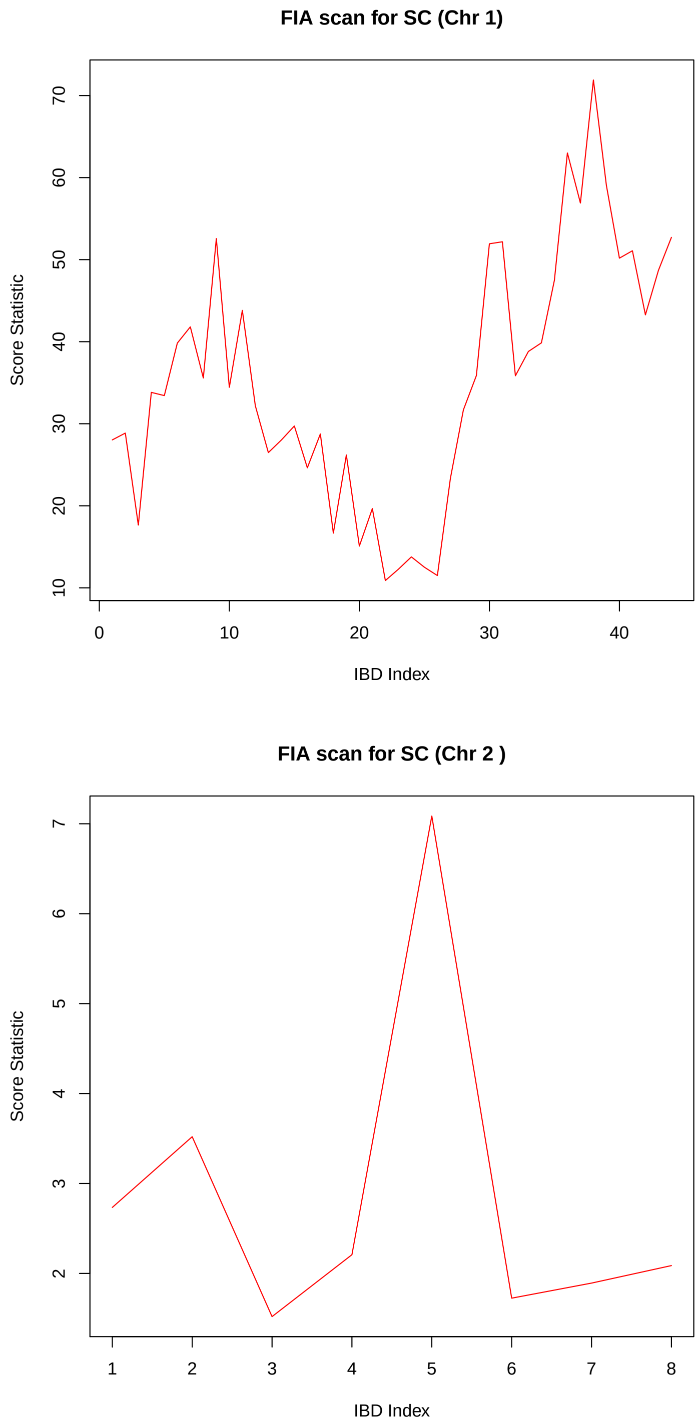

Figure 4: An example FIA QTL scan result.

```
Summary of the random effects estimates:

      Estimate Std. Error
Z.494   0.0169     0.0543
Z.495  -0.0294     0.0432
Z.496   0.0328     0.0522
...


----------------
DISPERSION MODEL
----------------

NOTE: h-likelihood estimates through EQL can be biased.

Dispersion parameter for the mean model:
[1] 0.1305672

Model estimates for the dispersion term:

Link = log

Effects:
  Estimate Std. Error
   -2.0359     0.0653

Dispersion = 1 is used in Gamma model on deviances to
calculate the standard error(s).

Dispersion parameter for the random effects:
[1] 0.003886 0.003324

Dispersion model for the random effects:

Link = log

Effects:
.|Random1
  Estimate Std. Error
   -5.5504     0.4263

.|Random2
  Estimate Std. Error
   -5.7065     0.4383

Dispersion = 1 is used in Gamma model on deviances to
calculate the standard error(s).
```

```
EQL estimation converged in 9 iterations.
```

We hope that this tutorial has been useful and our package facilitates your research. We are happy to receive any suggestions and will do our best to improve MAPfastR in response to user feedback. Thank you!

END

Alternative installation options

The simplest procedure for installing MAPfastR and all the required binaries is to use the command:

```
install.packages('MAPfastR',
 repos='http://www.computationalgenetics.se/MAPfastR')
```

However, it is necessary to build any R package from source on the Linux operating system (Note that R version 2.15.3 is supported). Some users may want to follow this procedure on OS X and Windows. Use the following command to do so:

```
install.packages('MAPfastR',
 repos = c('http://CRAN.R-project.org', 'http://R-Forge.R-project.org'),
 type='source')
```

Note that two repositories are called. This happens because the MAPfastR package and the following dependencies are located on R-Forge: cnf2freq and cnf2freqibdtracer. The dependencies are located on CRAN and include hglm, lattice, MASS, Rcpp, noia, sfsmisc, snow, snowfall, dglm, statmod, and Matrix. For Windows the required compilers are automatically installed with Rtools. The required compilers on OS X are installed with Xcode. Submit any questions to https://groups.google.com/d/forum/mapfastr

Bibliography

[1] L Crooks, C Nettelblad, and Ö Carlborg. An improved method for estimating chromosomal line origin in qtl analysis of crosses between outbred lines. *G3: Genes Genomes Genetics*, (1: 57-64), 2011.

[2] L Rönnegård, F Besnier, and Ö Carlborg. An improved method for qtl detection and identification of within-line segregation in f2 intercross designs. *Genetics*, (178), 2008.

[3] Ö Carlborg and L Andersson. The use of randomization testing for detection of multiple epistatic qtl. *Genetical Research*, (79), 2002.

[4] RM Nelson, C Nettelblad, L Crooks, ME Pettersson, F Besnier, X Shen, J Álvarez Castro, L Rönnegård, W Ek, Z Sheng, M Kierczak, S Holmgren, and Ö Carlborg. Mapfastr: Qtl mapping in outbred line crosses. *Bioinformatics*, 2012.

[5] C Nettelblad, S Holmgren, L Crooks, and Ö Carlborg. cnf2freq: Efficient determination of genotype and haplotype probabilities in outbred populations using markov models. In *Bioinformatics and Computational Biology*, number 5462 in Lecture notes in Bioinformatics. Springer, 2009.

[6] J Alvarez-Castro and Ö Carlborg. A general model for functional and statistical epistasis and its application in qtl analysis. *Genetics*, (176), 2007.

[7] J Alvarez-Castro, A Le Rouzic, and Ö Carlborg. How to perform meaningful estimates of genetic effects. *PLOS Genetics*, (4), 2008.
